# Anesthetic Management of a Broken Tracheostomy Tube Stem as a Foreign Body in the Trachea: A Case Report

**DOI:** 10.7759/cureus.53254

**Published:** 2024-01-30

**Authors:** Nisha Jain, Yudhyavir Singh, Abhishek Singh

**Affiliations:** 1 Anesthesiology, Pain Medicine & Critical Care, All India Institute of Medical Sciences, New Delhi, IND

**Keywords:** foreign bodies, pediatric airway emergency, supraglottic airway device, difficult airway management, tracheostomy complications

## Abstract

Tracheostomy is commonly performed in patients to secure the airway. There are known early and late complications related to tracheostomy. Few case reports have described the rupture of the stem of the tracheostomy tube and its migration into the tracheobronchial tree. Here we describe a pediatric case with a broken stem of the tracheostomy tube in the trachea, acting as a foreign body and causing mild respiratory distress. The patient was successfully managed with neck exploration under general anesthesia using a supraglottic airway device in low-resource settings. In addition, we have described the potential problems that may be faced while managing the airway of such patients and how to deal with these complications.

## Introduction

Emergency or elective surgical tracheostomy is commonly performed to secure the airway and for patients requiring prolonged mechanical ventilation. Approximately 20% of patients are discharged with a tracheostomy tube (TT) in situ [1]. Later, these cases are called up for regular follow-up for primary pathology in the outpatient department and to change the TT. There are known early and late complications related to tracheostomy. A broken tube and its unexpected presentation as a foreign body are two rare complications. We describe the management of one such case that required good clinical judgment and skilled airway management in emergency scenarios with limited resources.

## Case presentation

**Figure 1:**
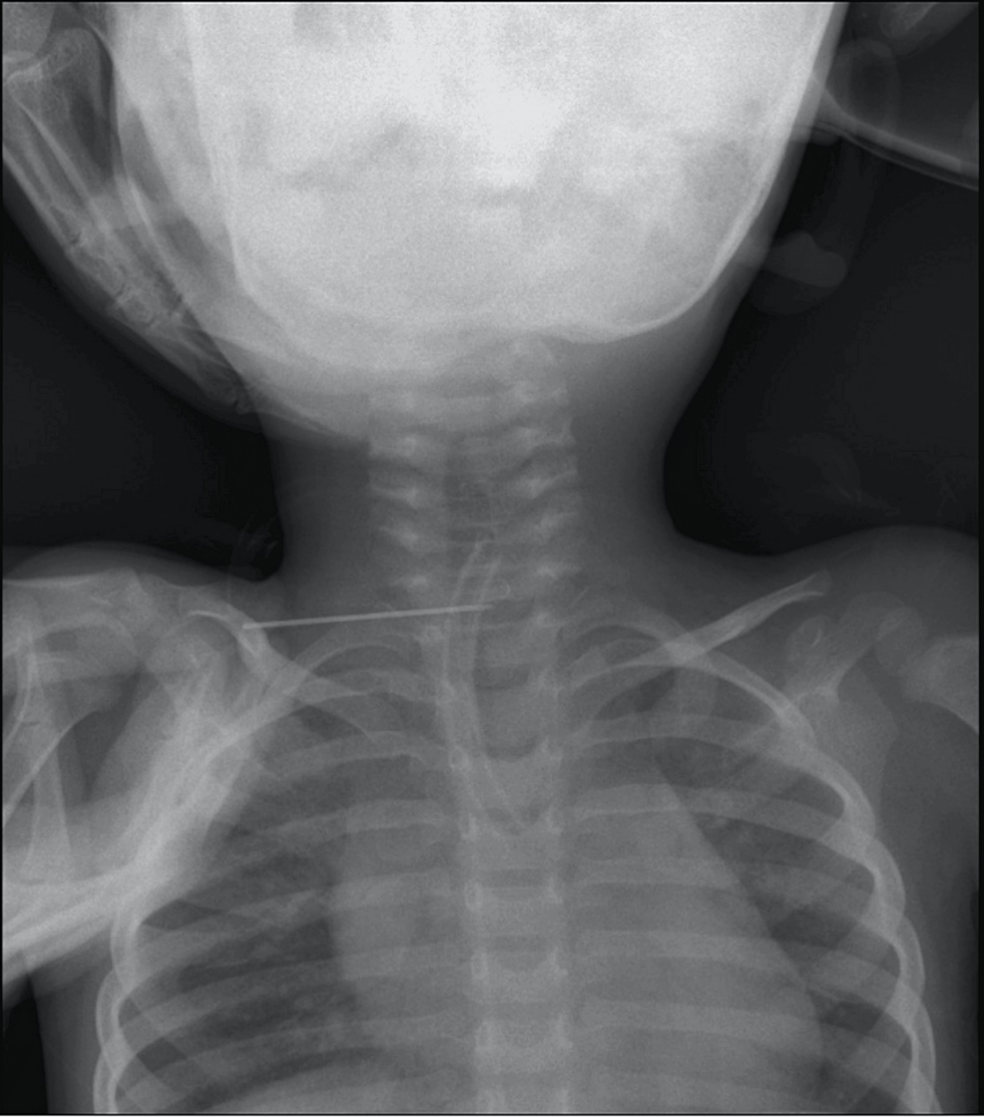
The presence of a broken stem of the tracheostomy tube in the trachea

A three-year-old, 10-kg tracheostomized child who was a follow-up case of frontotemporoparietal craniotomy was brought to the outpatient department of the trauma center for regular follow-up. On presentation, the child was coughing and was in minimal respiratory distress, which was unnoticed by the parents. He was hemodynamically stable with mild tachypnea and tachycardia. His room air oxygen saturation was 96-97%. When the TT strap was untied, the neck plate remained separated outside, and the stem of the broken TT went inside the airway. Bilateral air entry was present during auscultation, indicating that the foreign body did not completely obstruct the airway. Bedside chest X-ray revealed the stem of the TT in the trachea (Figure 1).

Because of the unavailability of rigid or flexible bronchoscopes in emergency settings, we decided to conduct neck exploration inside the operating room (OT). The patient was wheeled into the OT after obtaining informed, written parental consent. The American Society of Anesthesiologists standard monitors were used. The patient was maintained on spontaneous respiration with 100% oxygen using the Jackson-Rees (JR) circuit. Intravenous (IV) ketamine (10 mg) was administered via a 24G cannula, which was taken in the emergency department (ED). Supraglottic airway I-GEL size 2 was inserted, and sevoflurane was used for maintenance with spontaneous breathing. Endotracheal intubation was avoided because it could push the foreign body into the lower tracheobronchial tree, resulting in complete airway obstruction. Maintenance of spontaneous breathing with IV ketamine and second-generation supraglottic airways is the safest approach in these scenarios. The volatile agent sevoflurane can also be used without losing spontaneous breathing. The patient was hemodynamically stable throughout the procedure, with peripheral blood oxygen saturation between 99% and 100%.

**Figure 2:**
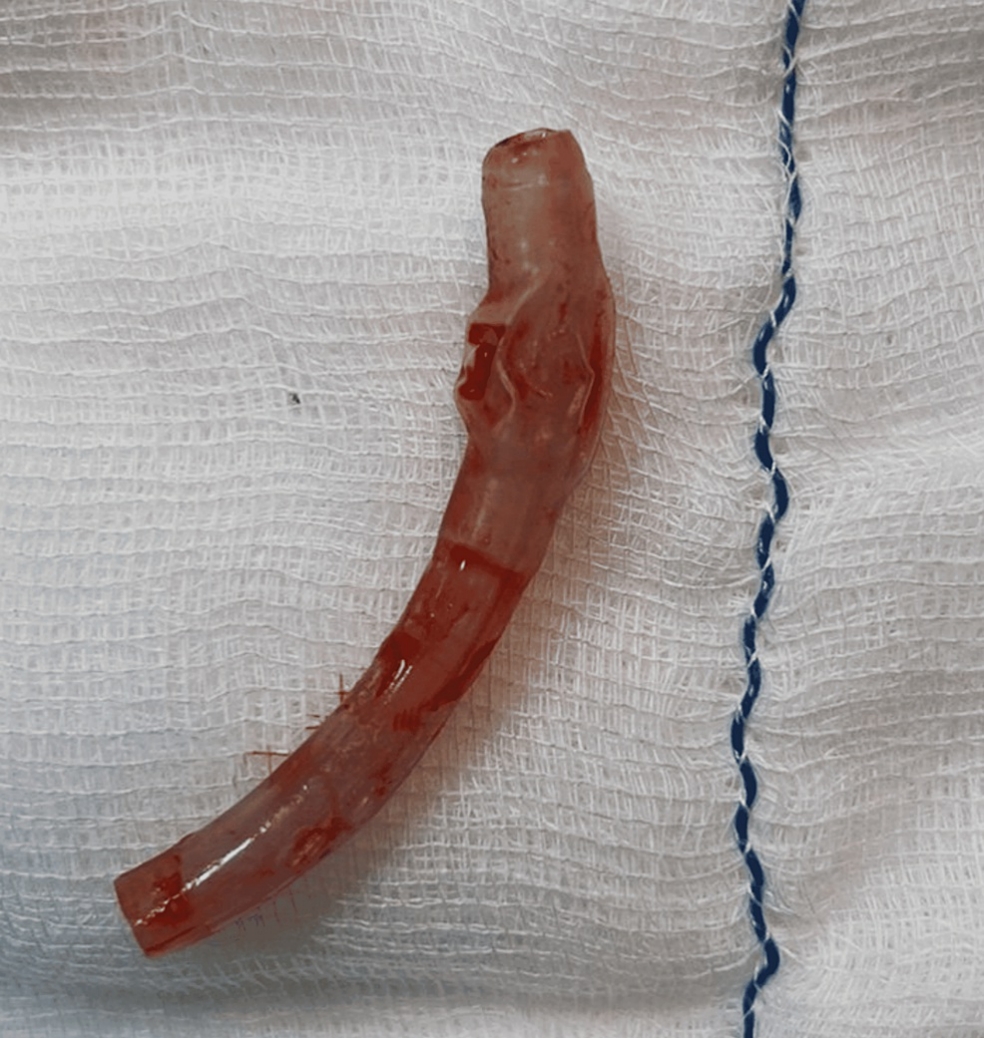
A broken stem of the tracheostomy tube extracted from the patient's airway

After positioning for surgical neck exploration, the patient suddenly became apneic with neck extension. Clinical judgment to rule out the cause of apnea and its management in such time-constrained conditions is of paramount importance. The extension of the neck brought one end of the in situ stem of the foreign TT close to the tracheal wall, which led to complete airway obstruction. After a neutral head position, spontaneous breathing was restored. Therefore, excessive neck extension should be avoided, and anesthesia providers should be vigilant in diagnosing the catastrophe. Successful removal of the foreign body was performed via neck exploration (Figure 2), and the patient was tracheostomized with a new 4-mm cuffed TT. Postoperatively, the patient was shifted to the ICU for observation and discharged after 24 hours with TT in situ.

## Discussion

The aspiration of broken parts of the TT was first reported by Bassoe and Boe in 1960 [2]. Since then, various similar cases and their management have been reported in the literature [3,4,5]. There are various available TTs, ranging from metal to polyvinyl chloride (PVC) or silicone. Preferred pediatric TTs are generally nonmetallic plastic tubes because of their low resistance, mucous adherence, and smooth surface [6].

Fracture at the junction of the stem and neck plate of the TT is the most common site [6]. The possible reasons for the fracture include the usage of the same TT, alkaline bronchial secretions, tissue reactions, repeated cleaning and sterilization, aging of the tube, and manufacturing defects [6]. The pediatric age group between one and three years is particularly at risk because of increased mobility, curiosity, and decreased parental supervision. The presenting spectrum of a tracheobronchial foreign body can be wide, ranging from minimal symptoms to respiratory failure.

A literature review has already concluded that bronchoscopic removal is the first line of management for aspirated tracheobronchial foreign bodies. A review by Lynrah et al. on fractured TT foreign bodies in three cases concluded that one patient was successfully managed with bronchoscopic removal of the fractured tube. The other patient succumbed to asphyxia before intervention, and the third patient had supratubal tracheal stenosis, which was managed by tracheoscopy through tracheostomy [7]. Gaurang Singhal et al. reported the successful retrieval of an aspirated fractured TT from the tracheobronchial tree using rigid bronchoscopy [6]. Piromachai et al. reviewed 20 cases and concluded that fracture of the metallic tube was more common than that of nonmetallic TTs. The trachea and the right main bronchus were the most common dislodgement sites [8]. Similar findings were reported by Parida et al. in a review of eight cases [9].

In this study, it was a PVC-type TT that was dislodged in the trachea. The patient presented with minimal respiratory distress, and the airway was secured with the second-generation supraglottic airway I-gel with the maintenance of spontaneous respiration. Successful removal of the foreign body was performed by neck exploration.

## Conclusions

Breakage of the TT and aspiration of broken parts into the tracheobronchial tree are life-threatening but avoidable complications. We report a pediatric case of a broken TT stem that remained in the trachea but was unnoticed by the parents. Proper care and high vigilance with regular suction and cleaning of the TT are of great clinical importance. Educating parents about the general care and related complications of TT in pediatric tracheostomized patients can reduce the incidence of such complications. Early diagnosis and management of such cases with skillful airway management in limited resource settings can significantly reduce morbidity and mortality.
